# Charge Transport and Glassy Dynamics in Blends Based on 1-Butyl-3-vinylbenzylimidazolium Bis(trifluoromethanesulfonyl)imide Ionic Liquid and the Corresponding Polymer

**DOI:** 10.3390/polym14122423

**Published:** 2022-06-15

**Authors:** Maxi Hoffmann, Ciprian Iacob, Gina Kaysan, Mira Simmler, Hermann Nirschl, Gisela Guthausen, Manfred Wilhelm

**Affiliations:** 1 Karlsruhe Institute of Technology (KIT), 76131 Karlsruhe, Germany; maxi.hoffmann@kit.edu (M.H.); gina.kaysan@kit.edu (G.K.); mira.simmler@kit.edu (M.S.); hermann.nirschl@kit.edu (H.N.); gisela.guthausen@kit.edu (G.G.); 2 National Research and Development Institute for Cryogenic and Isotopic Technologies (ICSI), 240050 Ramnicu Valcea, Romania

**Keywords:** ionic liquids, imidazolium, ionic liquid blends, rheology, broadband dielectric spectroscopy, PFG-NMR, charge transport

## Abstract

Charge transport, diffusion properties, and glassy dynamics of blends of imidazolium-based ionic liquid (IL) and the corresponding polymer (polyIL) were examined by Pulsed-Field-Gradient Nuclear Magnetic Resonance (PFG-NMR) and rheology coupled with broadband dielectric spectroscopy (rheo-BDS). We found that the mechanical storage modulus $\left(G^{\prime }\right)$ increases with an increasing amount of polyIL and $G^{\prime }$ is a factor of 10,000 higher for the polyIL compared to the monomer ($G_{IL}^{\prime }$= 7.5 Pa at 100 rad s^−1^ and 298 K). Furthermore, the ionic conductivity $\left(\sigma_{0}\right)$ of the IL is a factor 1000 higher than its value for the polymerized monomer with $3.4\times 10^{-4}$ S cm^−1^ at 298 K. Additionally, we found the Haven Ratio ($H_{R})$ obtained through PFG-NMR and BDS measurements to be constant around a value of 1.4 for the IL and blends with 30 wt% and 70 wt% polyIL. These results show that blending of the components does not have a strong impact on the charge transport compared to the charge transport in the pure IL at room temperature, but blending results in substantial modifications of the mechanical properties. Furthermore, it is highlighted that the increase in $\sigma_{0}$ might be attributed to the addition of a more mobile phase, which also possibly reduces ion-ion correlations in the polyIL.

## 1. Introduction

The unique properties of polymerized ionic liquids (polyILs), which combine the high ionic conductivity of ionic liquids (ILs) with the high mechanical moduli of polymers, have made them very attractive materials for electrochemical devices, e.g., batteries, fuel, and solar cells, among others [1,2,3,4]. Compared to classical liquid-electrolytes, solid-polymer electrolytes used in lithium-ion batteries (LIBs) provide excellent mechanical properties and thermal stability without leakage, flammability, or volatility [5,6,7,8]. Moreover, most applications of polyILs would benefit from high ionic conductivity while maintaining mechanical integrity.

To achieve high ionic conductivities in these systems, polyILs with lower glass transition temperatures have been synthesized. Unfortunately, higher ionic conductivities were achieved while the mechanical properties deteriorated.

To overcome this issue, the polyIL investigated here takes advantage of the decoupling of charge transport from molecular segmental motion below the polymer’s glass transition temperature ($T_{g})$ of 281 K. In the classical theory of ion conduction, the coupled segmental relaxation and charge transport are related as follows [9]:

(1) $$\sigma_{0}\tau_{\alpha }T=constant$$

where $\sigma_{0}$ is the ionic conductivity, $\tau_{\alpha }$ the segmental relaxation time, and T the absolute temperature. Equation (1) implies that ion transport is only possible via segmental motion, which leads to a tremendous drop of $\sigma_{0}$ below $T_{g}$ due to a drastic increase in $\tau_{\alpha }$ below $T_{g}$. Above $T_{g}$ the temperature dependence of $\sigma_{0}$ usually follows a Vogel–Fulcher–Tammann behavior, whereas below $T_{g}$ an Arrhenius dependence is found [10]. Different studies revealed that a decoupling of the two processes can be achieved using rigid polymers [11,12]. To gain a deeper knowledge, different authors set a focus on the investigation of the decoupling of charge transport from ion motion [11,13,14,15,16]. Iacob et al. offered insights into the impact of alkyl chain lengths and anion sizes on the $T_{g}$-independent ionic conductivity [17,18]. They concluded that the $T_{g}$-independent ionic conductivity decreases more than one decade by increasing the alkyl side chain length from ethyl (*n* = 2) to octyl (*n* = 8). Moreover, $\sigma_{0}$ was shown to increase for smaller anions, and both parameters did not seem to have an impact on the decoupling. The influence of the degree of decoupling on the ionic conductivity below $T_{g}$ was investigated by Wang et al. and Fan et al. [16,19]. They found an increasing conductivity with an increasing fragility (m), which was reached by incorporating especially rigid structures into already existing solid polymer electrolytes.

(2) $$m={\frac{d\;\log\;\tau_{\alpha }}{d\left(\frac{T_{g}}{T}\right)}|}_{T=T_{g}}$$

Despite this, the conductivity of pure polyILs is still not sufficient for practical applications at ambient temperatures, where a typical goal would be to reach an ionic conductivity of $1\times 10^{-3}$ S cm^−1^ or higher. To overcome this drawback, the addition of the corresponding monomer as a plasticizer was studied to accelerate the dynamics and increase the number of ions, thereby enhancing the ionic conductivity. Recent publications demonstrate the combination of highly conductive ILs with a host polymer as a gel polymer electrolyte (GPE) [20,21,22,23,24]. For example, De Anastro et al. photopolymerized poly(ethylene glycol)diacrylate (PEGDA) combined with an IL and the corresponding lithium salt. The obtained films were flexible and showed conductivities only slightly lower than those of the pure IL of around $6.5\times 10^{-3}\;$ S cm^−1^ at 323 K [21]. Opposing this, Wang et al. studied high lithium-concentration phosphonium ILs in poly(diallyldimethylammonium) bis(trifluoromethanesulfonyl)imide ([PDADMA]TFSI) and achieved ionic conductivities of up to $8.6\times 10^{-5}$ S cm^−1^ and an improved mechanical strength with an elastic shear modulus of $G^{\prime }=$ $4.4\times 10^{6}\;$ Pa by adding Al_2_O_3_ nanoparticles [20]. Other matrices were investigated by Sen et al. who crosslinked poly-1-butyl-3-vinylbenzylimidazolium bis(trifluoromethanesulfonyl)imide (poly[VBBI]TFSI, charged host polymer) and poly-1-(4-vinylbenzyl)imidazole (neutral host polymer) in combination with different ILs such as diethylmethylammonium trifluoromethanesulfonate ([DEMA]TFSI), and diethylmethylammonium bis(trifluoromethanesulfonyl)imide ([DMA]TFSI). For both host polymers, a decrease in thermal stability compared to the neat IL was observed, while the ionic conductivity of the charged polymer host was higher by a factor of up to six compared to the neutral polymer [22].

However, when ILs are used in combination with polymers as electrolytes for the perspective of increasing the ionic conductivities in energy storage applications, leakage may still be a problem because the ILs may only weakly/partially interact with the polymer network. To overcome this, Põhago-Esko et al. examined blends of photopolymerized 1-[*n*-(methacryloyloxy)alkyl]-3-methylimidazolium bromides (n=2, 6, 7, or 10) with 1-ethyl-3-methylimidazolium tetrafluoroborate ([EMIM]BF4) as IL additive [25]. The blends maintained solidity up to 40% *v*/*v* of IL content, with ionic conductivities of approximately $10^{-4}$ S cm^−1^. In addition, Marcilla et al. blended poly(1-vinyl-ethyl-imidazolium) having different counter-anions (TFSI^−^, BF_4_^−^, and Br^−^) with 1-butyl-3-methylimidazolium bis(trifluoromethanesulfonimide) ([BMIM]TFSI), 1-butyl-3-methylimidazolium tetrafluoroborate ([BMIM]${\mathrm{BF}}_{4}$) and 1-butyl-3-methylimidazolium bromide ([BMIM]Br) as ILs [26]. They observed ionic conductivities in the range of $10^{-5}$ to $10^{-2}$ S cm^−1^ at room temperature, which increased with IL content. Furthermore, molecular simulations were conducted by Mogurampelly et al. to study the ion transport in blends of ILs and their polymers. They investigated mixtures of 1-butyl-3-methyl-imidazolium hexafluorophosphate and poly(1-butyl-3-vinylimidazolium) hexafluorophosphate and suggested the existence of a similar charge transport mechanism controlled by intra- and intermolecular hopping of ions, as observed in their earlier reports of polyILs [27,28]. Additionally, they found stronger interactions between the anions and the polymer cations than with the IL-cations. They observed that the non-monotonic behavior of the ionic conductivities in the blends of polyILs and ILs was controlled by the interplay between ion mobility, decoupling of ion transport from the segmental motion of the polymer, and the number of free ions, which reveals the rich properties in these materials.

Furthermore, the mobility of the ions was investigated by pulsed-field gradient nuclear magnetic resonance spectroscopy (PFG-NMR) [29]. PFG-NMR allows for the determination of the translational dynamics and diffusion coefficients of moieties [30,31,32]. To relate the diffusion coefficients to the ionic conductivities measured by broadband dielectric spectroscopy (BDS), the diffusion coefficient of the conduction process ($D_{\sigma })$ can be calculated according to the Nernst-Einstein equation, as follows [33]:

(3) $$D_{\sigma }=\frac{n_{0}q^{2}}{kT}\sigma_{0}$$

where $n_{0}$ is the number density of the ions, q the elementary charge, k the Boltzmann constant, and T the absolute temperature. The ionic conductivity $\sigma_{0}$ is determined from the frequency-independent plateau in the real part of the conductivity $\left(\sigma^{\prime }\left(\nu \right)\right)$ via BDS. The diffusion coefficient obtained by PFG-NMR is often reported to be larger than the one obtained by BDS [34]. This difference, or rather the related ratio, is considered to originate from the presence of ion pairs and aggregates, which do not contribute to the process of charge transport. The ratio of the two diffusion coefficients can be quantitatively described by the Haven ratio $\left(H_{R}\right)$ as follows:

(4) $$H_{R}=\frac{D_{mean}}{D_{\sigma }}=\frac{n_{0}q^{2}}{kT}\times \frac{D_{mean}}{\sigma_{0}}\;$$

where $D_{mean}$ corresponds to the mean diffusion coefficient $D_{mean}=(D_{anion}+D_{cation})/2$. If $H_{R}$ exceeds 1 it indicates that ion pairs have formed; these ion pairs do not contribute to the charge transport and are therefore neglected by BDS measurements. Typical values of $H_{R}$ are around 1.4 for ILs, indicating a low aggregate concentration, but higher values (e.g., $H_{R}=$ 5), resulting in correlated ion motion in the materials, have been found for polyILs [34,35,36,37].

Despite the numerous contributions, most of the studies focus mainly on the application of polyIL/IL blends in energy storage. There is still a lack of understanding of the mechanism of charge transport and dynamics in these mixtures. Therefore, in this study, we present a systematic investigation of different blends of poly[VBBI]TFSI and the respective monomer to create thermally stable polymer electrolytes, avoiding phase separation, and enhancing the ionic conductivity of the final materials. The objective is to better understand the impact of an IL on the polymer host in terms of charge transport and segmental relaxation. The blends of the self-synthesized polyIL and the related IL are investigated by a unique combination of rheology and broadband dielectric spectroscopy to examine the influence of the IL on mechanical stability and ionic conductivity. Furthermore, we take a closer look at the diffusion process in the IL as well as at two blends to better understand the mechanism and efficiency of charge transport in these blends.

## 2. Materials and Methods

### 2.1. Materials

4-Vinylbenzylchlorid (90%, stabilized with 500 ppm tert-butylcatechol, Sigma Aldrich), 1-butylimidazole (>99%, Alfa Aesar, Haverhill, MA, USA), acetonitrile (>99.7%, VWR, Radnor, PA, USA), 2,6-di-tert-butyl-4-methylphenol (BHT, for synthesis, Merck, Darmstadt, Germany), diethyl ether (99.5%, Acros Organics, stabilized with BHT), lithium bis(trifluoromethanesulfonyl)imide (LiTFSI, >99%, Acros Organics, Waltham, MA, USA), acetone (>99.5%, Acros Organics), *n*-pentane (>99%, VWR), 2,2′-Azobis(2-methylpropionitrile) (AIBN, 98%, Merck), dimethylformamide (DMF, 99.8%, Acros Organics), methanol (>99%, Acros Organics), and tetrahydrofuran (THF, 99%, Acros Organics) were used as received.

### 2.2. Synthesis

**Scheme 1:**
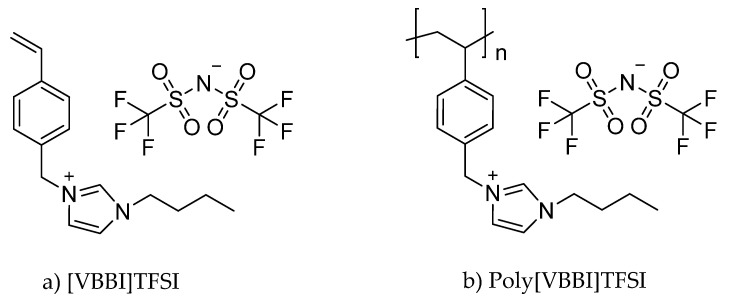
Chemical structure of (**a**) 1-butyl-3-vinylbenzylimidazolium bis(trifluoromethanesulfonyl)imide ([VBBI]TFSI) and (**b**) the corresponding polymer poly[VBBI]TFSI.

The synthesis of poly[VBBI]TFSI was adapted from Tang et al. [38]. The IL monomer was prepared by anion exchange and polymerized by radical polymerization. More details about the synthesis can be found in the supplementary information (SI). The structures of the synthesized IL monomer and its polymer are displayed in Scheme 1 and their ^1^H-Nuclear Magnetic Resonance (NMR) spectra are shown in Figures S1 and S2.

### 2.3. Methods

Table 1 displays an overview of the investigated samples, starting with the polyIL, its blends with the corresponding IL, and the pure IL. The table displays the corresponding glass transition temperature $\left(T_{g}\right)$ from differential scanning calorimetry (DSC) measurements, the ionic conductivity $\left(\sigma_{0}\right),$ and the storage modulus $\left(G^{\prime }\right)$ at room temperature (RT). 

#### 2.3.1. Size Exclusion Chromatography (SEC)

The number and weight average molecular weights $(M_{n}$ and $M_{w})$ were determined by SEC. The Agilent system was equipped with an iso-pump (1100 series), a UV detector (1200 series), a differential refractive index (DRI) detector (1200 series), a PSS SLD 7000 MALLS detector, and two PSS SDV Lux 8 × 300 mm columns. The column materials possessed a mean effective pore size diameter of $10^{3}$ Å and $10^{5}$ Å, respectively. For the analysis, the polymer was dissolved in a solution of 10 mM 1-butylimidazole and 10 mM LiTFSI in tetrahydrofuran (THF), which was also used as eluent, and was analyzed at 23 °C with a flow rate of 1 mL min^−1^ [39]. The molecular weight was obtained using the MALLS detector and therefore, the refractive index increment (dn/dc) was determined with a refractometer dn/dc 2010 from PSS (Mainz, Germany) using ten different solutions from 1 g L^−1^ to 10 g L^−1^ polyIL in a solution of 10 mM 1-butylimidazole and 10 mM LiTFSI in THF (dn/dc =0.1027 mL ·g^−1^). Prior to the measurements, the different solutions were filtered two times through a 0.2 μm PVDF membrane. The molecular weight of the polyIL was $M_{n}=57,000$ g mol^−1^, $M_{w}=129,000$ g mol^−1^, and Ð = 2.25.

#### 2.3.2. Differential Scanning Calorimetry (DSC)

DSC measurements were performed under nitrogen using a DSC Q200 (TA Instruments, Milford, CT, USA) with a heating rate of 10 K min^−1^. Two heating cycles were run while the $T_{g}$ of the samples was determined from the inflection point of the second heating cycle. The DSC curves of all samples are displayed in Figure S3 in the SI.

#### 2.3.3. Wide-Angle X-ray Scattering (WAXS)

To investigate the change in the morphology, WAXS measurements were performed on a Xeuss 2.0 (Xenocs, Grenoble, France) system with a point collimated X-ray beam ($0.8\times 0.8\;{\mathrm{mm}}^{2}$), monochromatized on Cu-K-alpha (λ=0.154 nm), and focused on the detection plane at a sample detector distance of 80 mm. The samples were transferred into a gel capsule (diameter: 4 mm, thickness: 1 mm) with polyimide foils (Kapton) as windows. The scattering intensity I(q) was measured for 1800 s and detected by a 2D-detector (PILATUS 100K-S, Dectris, Baden, Switzerland). The Kapton background was subtracted from the data after normalization to the primary beam. The WAXS data is displayed in Figure S4 in the SI.

#### 2.3.4. Rheological and Dielectric Measurements

For Broadband Dielectric Spectroscopy (BDS) and rheological measurements, typically 100 mg of sample was prepared. The respective amounts of IL and polyIL were dissolved in 0.3 mL of acetonitrile and stirred overnight to prepare homogeneous solutions. The sample was injected into a plastic mold and placed in a fume hood for 2 h. Then, the sample was dried under reduced pressure (approx. $10^{-3}$ mbar) at 323 K (50 °C) for 48 h. Comparison of NMR measurements of the samples with the pure solvent revealed a remaining water content of approximately 1%.

Rheological and dielectric measurements were performed on a unique rheo-BDS setup consisting of a strain-controlled ARES G2 rheometer (TA Instruments, Milford, CT, USA) and an ALPHA-Analyzer (Novocontrol, Montabaur, Germany) [40]. Oscillatory shear measurements were performed using a homemade 8 mm parallel plate-plate geometry with ceramic insulation between the parallel plates and the fixture and it was connected to the ALPHA-Analyzer by a highly temperature resistant wire. Additional information on the geometry and the setup can be found elsewhere [10,40]. Small amplitude oscillatory shear (SAOS) measurements were performed in an angular frequency range from 0.1 to 100 rad s^−1^ at different temperatures and the master curves were obtained using the time-temperature superposition (TTS) principle reduced to a reference temperature $\left(T_{ref}\right)$ of 273 K. Dielectric measurements were performed between 0.1 Hz and 1 MHz by applying a voltage of 0.1 V at a distance between the plates of about 1 mm in order to avoid any electrochemical process at the interface between electrolyte and electrode. Before the measurements, the samples were annealed at 373 K for 15 min and allowed to equilibrate at each temperature for 10 min.

#### 2.3.5. Pulsed-Field Gradient Nuclear Magnetic Resonance (PFG-NMR)

A 400 MHz spectrometer (BRUKER Avance Neo 400 WB) equipped with a DiffBB probe was used for the PFG-NMR measurements. Dry [VBBI]TFSI was directly placed in a conventional 5 mm NMR glass tube whereas the other samples were dissolved in 0.5 mL of acetonitrile overnight and placed into the NMR tubes. The tubes were dried at 60 °C under reduced pressure for at least 72 h. All tubes were filled approximately 1 cm in height to avoid fluidic convection. 

Diffusion coefficients for the cation and anion were obtained by ^1^H-NMR (cation) and ^19^F-NMR (anion) using a pulsed-field gradient stimulated echo (PFG-STE) sequence at different temperatures. Diffusion coefficients were calculated using the Stejskal–Tanner equation [29,41,42] by measuring the ratio *S*/*S*_0_ as follows:

(5) $$S=S_{0}\exp\left(-D\gamma^{2}g^{2}\delta^{2}\left(\Delta -\frac{\delta }{3}\right)\right)$$

where $S_{0}$ is the signal at g=0, D the translation diffusion coefficient, γ the gyromagnetic ratio, g the pulsed gradient amplitude, δ the duration of the gradient pulses, and Δ the diffusion time. The duration of the gradient pulses was set to δ=1.6 ms, the diffusion time to Δ=40 ms with an acquisition time of 4 ms, and g ∈[3,15] T/m depending on the temperature. The number of scans was set to 2 for all samples and the temperature was allowed to equilibrate for at least 30 min. As a reference substance, 200 μL of ethylene glycol was filled into the NMR tube and the sample temperature was deduced from the shift of the peaks according to Ammann et al. [43]. 

## 3. Results and Discussion

### 3.1. Rheological Measurements

**Figure 1:**
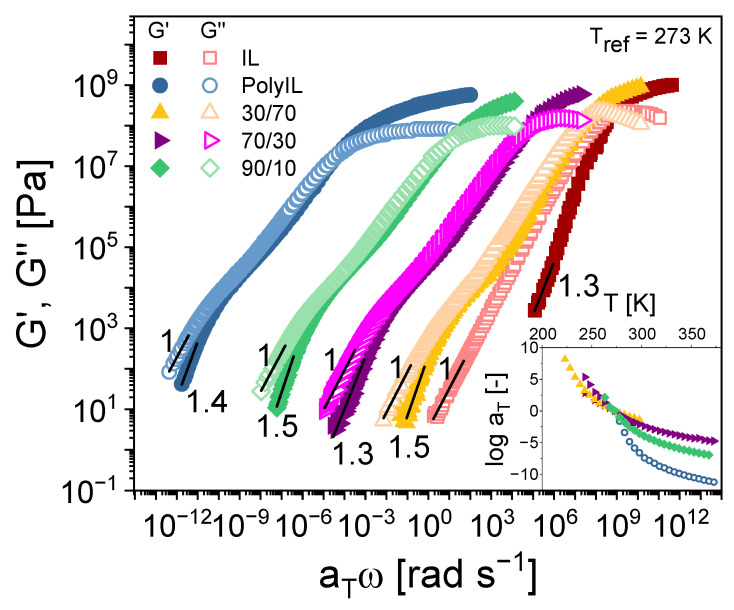
Rheological master curves of five samples (neat ionic liquid (IL), neat polymerized ionic liquid (polyIL), 30 wt% polyIL, 70 wt% polyIL, and 90 wt% polyIL) constructed using time-temperature superposition (TTS) at the indicated reference temperature. The inset displays the TTS shift factors of the sample for $T_{ref}=273$ K.

SAOS measurements were performed to determine the influence of the amount of IL on the mechanical properties. Figure 1 presents the master curves of the shear storage $\left(G^{\prime }\right)$ and loss modulus $\left(G^{\prime \prime }\right)$ of five selected samples constructed using the time-temperature superposition (TTS) principle at a reference temperature of $T_{ref}=273$ K including the respective shift factors in the inset, while the master curves of the remaining samples are displayed in Figure S7 in the SI. 

TTS was found to work well for all samples as confirmed by the $a_{T}$ shift factors. In the terminal regime $G^{\prime \prime }\propto \omega$ was observed while otherwise $G^{\prime }\propto \omega^{1.3–1.5}$. Each sample exhibited a crossover at high frequencies corresponding to the segmental relaxation (α-relaxation) at $T_{g}.$ Furthermore, the samples displayed neither a crossover of the moduli at low frequencies nor a rubber plateau, which led to the assumption that no effective polymer chain entanglements are present for $M_{n}=57,000$ g mol^−1^. As the wt% of IL increased, $G^{\prime }$ is observed to decrease by $10^{5}$ from 10 wt% IL to 90 wt% IL at 298 K and 1 rad s^−1^, thus yielding flowing samples with a $G^{\prime }$ of ca. 10 Pa for 90 wt% IL. This drastic decrease is caused by the IL acting as a plasticizer in the polyIL. 

### 3.2. Dielectric Properties 

**Figure 2:**
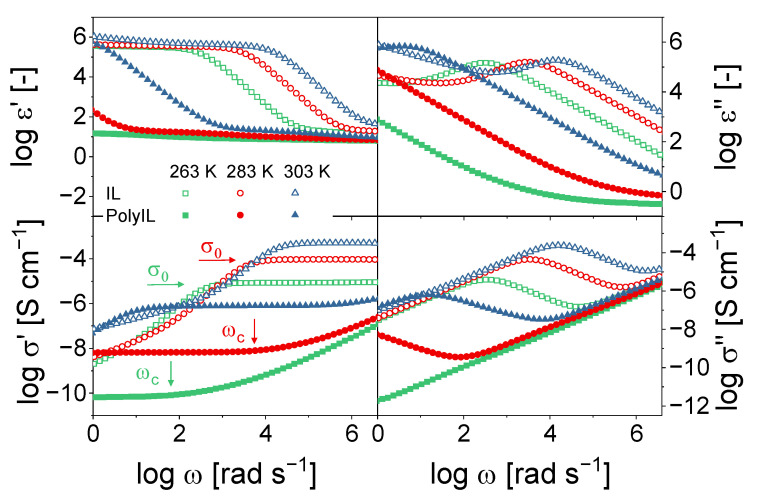
The real and imaginary part of the complex permittivity ($\epsilon^{\prime }$ and $\epsilon^{\prime \prime }$ ) and the real and imaginary part of the complex conductivity ($\sigma^{\prime }$ and $\sigma^{\prime \prime }$ ) as a function of the angular frequency at three representative temperatures for the IL, open symbols, and the polyIL, closed symbols. The ionic conductivity $\left(\sigma_{0}\right)$ and the rate of charge transport $\left(\omega_{c}\right)$ are indicated in the real part of the conductivity $\left(\sigma^{\prime }\right)$.

The dielectric raw data for the IL and polyIL as a function of the angular frequency at three different temperatures is displayed in Figure 2, where the complex permittivity ($\epsilon^{*})$ and complex conductivity ($\sigma^{*})$ are related as follows:

(6) $$\sigma^{*}\left(\omega ,\;T\right)=i\epsilon_{0}\omega \epsilon^{*}\left(\omega ,T\right)$$

with $\epsilon_{0}$ being the vacuum permittivity, ω the angular frequency, and T the absolute temperature.

The decrease in the low-frequency regime of the real part of the conductivity ($\sigma^{\prime })$ is assigned to electrode polarization, where ions accumulate at the electrodes leading to the blocking of the electrodes [44,45]. Furthermore, $\sigma_{0}$ and the rate of charge transport $\left(\omega_{c}\right)$, which are the two components of charge transport, are obtained from $\sigma^{\prime }$ as the independent frequency-plateau value and frequency at which $\sigma^{\prime }$ turns into a power law, respectively [46]. As the onset of the power law in $\sigma^{\prime }$ and therefore $\omega_{c}$ is hard to determine, the frequency of the maximum of the imaginary part of the electric modulus ($M^{\prime \prime }$), which is known to be almost the same as $\omega_{c}$ (Figure S6), is used [47,48]. The electric modulus ($M^{*})$ is related to $\epsilon^{*}$ and $\sigma^{*}$ as follows:

(7) $$M^{*}\left(\omega ,\;T\right)=\frac{1}{\epsilon^{*}\left(\omega ,\;T\right)}=\frac{i\epsilon_{0}\omega }{\sigma^{*}\left(\omega ,\;T\right)}$$

where $\epsilon_{0}$ is the vacuum permittivity, ω the angular frequency, and T the absolute temperature.

**Figure 3:**
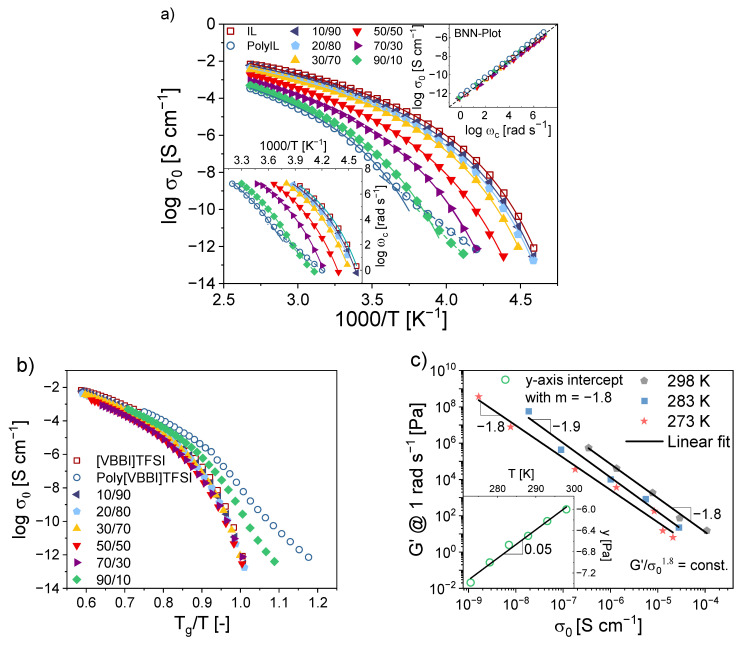
(**a**) Ionic conductivity ($\sigma_{0})$ as a function of the inverse temperature including VFT and Arrhenius fits (solid and dashed lines, respectively). The inset on the upper right shows $\sigma_{0}$ versus the rate of charge transport $\left(\omega_{c}\right)$. The Barton–Nakajima–Namikawa relationship (BNN) indicates that the mechanism of the charge transport is the same for all samples. The inset on the lower left displays the temperature dependence of $\omega_{c}$, which is the same as for $\sigma_{0}$. (**b**) $\sigma_{0}$ as a function of $T_{g}/T$ to suppress the influence of the different $T_{g}$ of the samples. (**c**) The correlation between the storage modulus $\left(G^{\prime }\right)$ at 1 rad s^−1^ and $\sigma_{0}$ at three temperatures is found to be linear to a good approximation. The inset in (**c**) shows the temperature dependence of the y-axis intersection of the linear fits from (**b**) using a slope of 1.8, which also shows a linear dependence on T.

In general, the observed ionic conductivity of the IL at 283 K is about 4 decades higher than that of the corresponding polyIL, which is due to the increased $T_{g}$ and reduced mobility of the polymeric form. Additionally, only the anions are taking part in the charge transport in the polyIL, resulting in a lower number of moving ions [36]. Consequently, the addition of IL to the polyIL led to increased ionic conductivities due to (1) an enhanced mobility of the polymer and monomer and (2) a higher number of available charges because of the more mobile cations in the IL (Figure 3a). The mobility of the ions is further enhanced at higher temperatures and is coupled to the segmental motion as represented by the Vogel–Fulcher–Tammann (VFT) behavior observed for $T>T_{g}$ [49]. The mobility is directly related to the ionic conductivity with the following:

(8) $$\sigma_{0}=\sigma_{\infty }\exp\left(-\frac{B}{T-T_{0}}\right)$$

where $\sigma_{\infty }$ is the conductivity at an infinitely high temperature, B is a fitting parameter related to the activation energy, and $T_{0}$ the Vogel temperature $\left(T_{0}\approx T_{g}-50\;K\right)$. The VFT equation can be mathematically transformed into the Williams–Landel–Ferry (WLF) equation as $T_{0}$ is related to $C_{2}$ at $T_{g}=T_{ref}.$ While most of the samples display the VFT behavior over the entire investigated temperature range, an Arrhenius behavior is observed for the polyIL and 90 wt% polyIL at $T<T_{g}$ as [50]

(9) $$\sigma_{0}=\sigma_{\infty }^{A}\exp\left(-\frac{E_{a}}{k_{B}T}\right)$$

where $\sigma_{\infty }^{A}$ is the conductivity at an infinitely high temperature for the Arrhenius behavior, $E_{a}$ is the activation energy, and $k_{B}$ is the Boltzmann constant. As the polymer is frozen below $T_{g}$, it is considered that only the mobile anions (see Scheme 1) contribute to the charge transport of the polyIL. In contrast, in the case of the blends, the cation of the IL also participates in charge transport. The charge transport is then controlled by different parameters, such as the anion size and polymer structure [35]. The corresponding activation energy for the diffusion process below $T_{g}$ is at 149 and 119 kJ mol^−1^ for 90 wt% polyIL and the pure polyIL, respectively (see Table 2), and is of the same magnitude as values found in the literature [18,35,51,52]. The transition between the VFT and Arrhenius behavior is found close to the $T_{g}$ of both samples as predicted in literature [36,51]. All corresponding fit parameters for the samples are summarized in Table 2. Furthermore, the same behavior is found for $\omega_{c}$ as displayed in the inset in the lower left of Figure 3a.

Correlating $\sigma_{0}$ with the storage modulus G′ as displayed on a log-log scale in Figure 3c, a linear dependency with a slope of −1.8 to −1.9 was observed for three temperatures (298 K, 283 K, and 273 K). With increasing temperature, fewer data points were available for $G^{\prime }$ at 1 rad s^−1^ because the sensitivity limit of the rheometer was reached. The inset in Figure 3c shows the temperature dependence of the y-axis intersection of the linear fits from Figure 3c using a slope of −1.8 and a linear dependence with a slope of 0.05 is observed. To the authors’ knowledge, the observed dependencies and scaling laws were not yet investigated for other blends of polyILs and ILs, and it remains unclear whether the observed slopes are more generally valid for other systems also.

The mechanism of charge transport can be described by the Barton–Nakajima–Namikawa relationship (BNN), $\sigma_{0}$ versus $\omega_{c}$, which is displayed in the inset in the upper right of Figure 3a. Here, the BNN plot illustrates that the mechanism of charge transport is the same for all materials and blends and takes place by hopping of the charge carriers as described by the random barrier model [18,53,54]. Mogurampelly et al. observed the same mechanism using atomistic molecular dynamics simulations [28]. Their results showed that over 90% of the hopping events take place along the polymer backbone by the formation and breaking of ion pairs. In our case, the ionic conductivity of the polyIL is slightly higher (factor 2) than the one of the IL at the same rate of charge transport, which suggests small differences in the efficiency of charge transport. To evaluate these, the different mobilities and thus the influence of the different glass transition temperatures of the samples are suppressed by scaling T to $T_{g}$ in Figure 3b. It stands out that the ionic conductivities of the 90 wt% polyIL and the neat polyIL are consistently higher compared to the other blends with coinciding $\sigma_{0}$-profiles. These two samples displayed a $6\times 10^{2}$ (90 wt%) and $1.7\times 10^{4}$ (pure polyIL) higher $\sigma_{0}$ at $T_{g}$ suggesting a more effective ion transport when scaled to $T_{g}$ most likely due to the decoupling of charge transport from segmental motion [55]. The decoupling is observed in $\sigma_{0}$ (Figure 3a) where the profiles of the 90 wt% polyIL and pure polyIL undergo a transition from the VFT to the Arrhenius behavior and therefore avoid the tremendous drop of $\sigma_{0}$ at $T_{g}.$ Below $T_{g}$ the polymer is frozen and only the anions account for the remaining charge transport [13,16,55].

**Figure 4:**
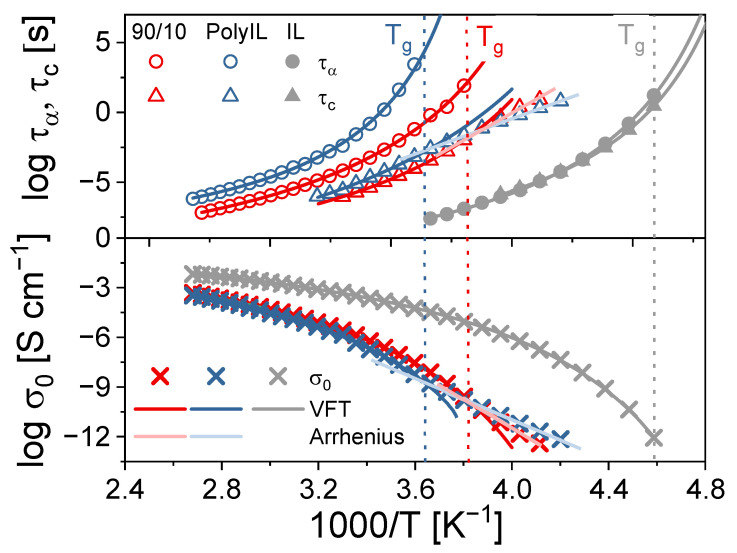
Temperature dependence of the segmental relaxation $\left(\tau_{\alpha }\right)$, the relaxation of charge transport $\left(\tau_{c}\right)$, and the ionic conductivity $\left(\sigma_{0}\right)$ of the IL, polyIL, and 90 wt% polyIL. Solid dark lines represent a VFT fit, whereas solid light lines describe an Arrhenius fit to the data. The dotted lines represent the respective $T_{g}$.

A closer look at the decoupling is taken by analyzing the segmental relaxation $\left(\tau_{\alpha }\right)$, the relaxation of charge transport $\left(\tau_{c}\right)$, and ionic conductivity ($\sigma_{0})$ of the polyIL and 90 wt% polyIL, see Figure 4.

**Figure 5:**
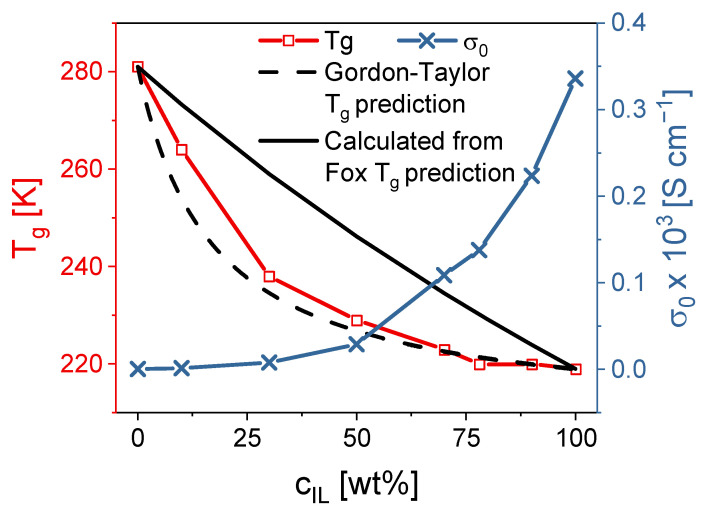
Influence of the ionic liquid concentration $\left(c_{IL}\right)$ on the $T_{g}$ (left y-axis, red) and ionic conductivity at 298 K (right y-axis, blue). The black dashed line represents the Gordon-Taylor prediction for $T_{g}$ with k=0.144, whereas the black line indicates calculated values from the Fox prediction for $T_{g}$.

While $\tau_{\alpha }$ and $\tau_{c}$ display VFT behavior above $T_{g}$, indicating the coupling of the two processes, the behavior is different at $T_{g}$:$\;\tau_{\alpha }$ increases towards infinity and $\tau_{c}$ transitions to Arrhenius behavior (light fit in Figure 4), as also observed for the ionic conductivity. Instead, both relaxation times are overlapping for the pure IL and show a drastic increase at $T_{g}$, confirming their strong coupling throughout the whole temperature range. The advantage of decoupling is only observed for one blend, meaning that IL at 30 wt% already suppresses this phenomenon. For these samples, the coinciding curves in Figure 3b reflect the strong dependence of $\sigma_{0}$ on $T_{g}$, which is further emphasized by the functional dependence of $T_{g}$ and $\sigma_{0}$ on the IL concentration shown in Figure 5. While $\sigma_{0}$ increases with the addition of IL, the $T_{g}$ is found to decrease. Both effects result from an enhanced mobility due to the increasing amount of IL. In general, the $T_{g}$ of a mixture of two components can be described by the Gordon-Taylor equation as follows [56]:

(10) $$T_{g}=\frac{w_{1}T_{g,1}+kw_{2}T_{g,2}}{w_{1}+kw_{2}}$$

where $w_{1}$ and $w_{2}$ are the weight fractions of the two components, $T_{g,1}$ and $T_{g,2}$ the respective glass transition temperatures, and k is the Gordon-Taylor constant (here: k=0.144). The Gordon-Taylor equation is an extension of the well-known Fox equation [57].

(11) $${\frac{1}{T_{g}}}_{\;}=\frac{w_{1}}{T_{g,1}}+\frac{w_{2}}{T_{g,2}}$$

For the fit, the measured $T_{g}$ of the IL and polyIL at 219 and 281 K, respectively, were used, and the unitless Gordon-Taylor constant of k=0.144 was obtained. Comparing the dependence of $T_{g}$ on $c_{IL}$ to that of the ionic conductivities, the same trend is observed and the highest conductivity of $3.4\times 10^{-4}$ S cm^−1^ is obtained for the pure IL possessing the lowest $T_{g}.$ Consequently, it reflects the importance of $T_{g}$ in the design of highly conductive materials as it limits $\sigma_{0}$.

**Figure 6:**
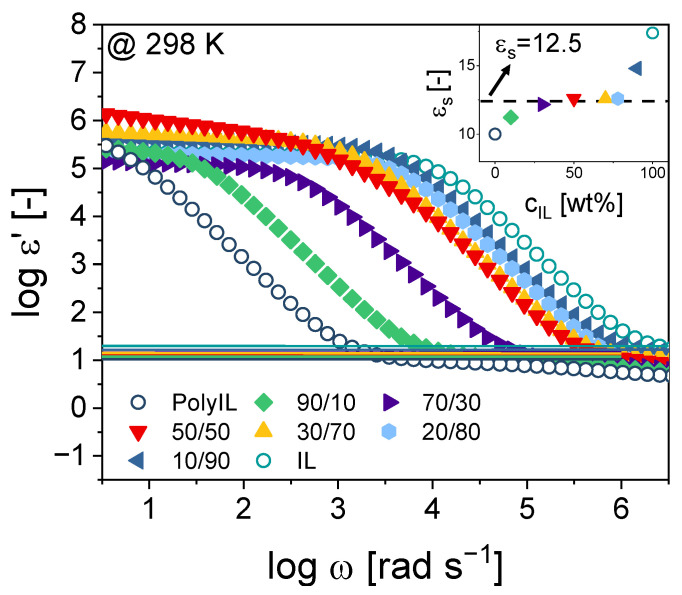
The real part of the permittivity $\left(\epsilon^{\prime }\right)$ and determination of the static dielectric constant $\left(\epsilon_{s}\right)$ at 298 K, plotted as solid line at the frequency plateau before electrode polarization sets in. The inset in the upper right displays $\epsilon_{s}$ as a function of the IL concentration where a plateau is observed at $\epsilon_{s}=12.5$.

However, $\sigma_{0}$ is influenced by not just the (de)coupling of the chain dynamics from the charge transport but also by the polarity of the medium, which offers insights about the accumulations of ions [58,59]. The polarity of a material is described by the static dielectric constant ($\epsilon_{s})$ as the low-frequency plateau before electrode polarization appears in $\epsilon^{\prime }\left(\omega \right)$ [34,59]. Figure 6 displays the frequency dependence of $\epsilon^{\prime }$ at 298 K for all samples, where the value of $\epsilon_{s}$ is found as a plateau before electrode polarization and is indicated as a solid line. 

At room temperature, $\epsilon_{s}$ = 10 was observed for the polyIL and $\epsilon_{s}$ = 17 for the IL, which is in the commonly observed range [60]. The addition of IL to the polyIL led to an increase in $\epsilon_{s}$ until a plateau was observed at 70 wt% IL with $\epsilon_{s}\;$= 12.5, which is highlighted by the dotted line in the inset in Figure 6. The inset shows the dependence of $\epsilon_{s}$ on the concentration of IL. As the behavior of $\epsilon_{s}$ is not linear, it reflects contributions from ‘other effects’ (e.g., interactions of polyIL and IL) beyond just the respective polarities of the samples. For low polarity samples, ($\epsilon_{s}<9$) clusters of aggregates and associated ions were observed by molecular simulation, leading to correlated ion movement [59,61]. These effects were shown to decrease with the increasing polarity of the samples. Furthermore, polymerization might restrict the ion motion, and also stronger anion and cation interactions are observed for polymerized samples compared to the IL [28]. Thus, our results indicate that already the addition of a low amount of IL results in weaker ionic interactions, which leads to an increased mobility of the ions and less correlated ion movement.

### 3.3. Diffusion Measurements

To further characterize the motion of the ions, ^1^H- and ^19^F-PFG-NMR were measured on three different samples (IL, 30 wt% polyIL, 70 wt% polyIL) at different temperatures, and the translational diffusion coefficients of the anions and cations were obtained. Temperature ranges were adjusted for each sample as severe peak broadening was observed. The peak broadening occurs because of restricted molecular mobility below $T_{g}$ and is reflected in the decreased transverse relaxation time ($T_{2}$). Diffusion coefficients of samples with higher contents of polyIL and pure polyIL could not be measured in the investigated temperature range.

**Figure 7:**
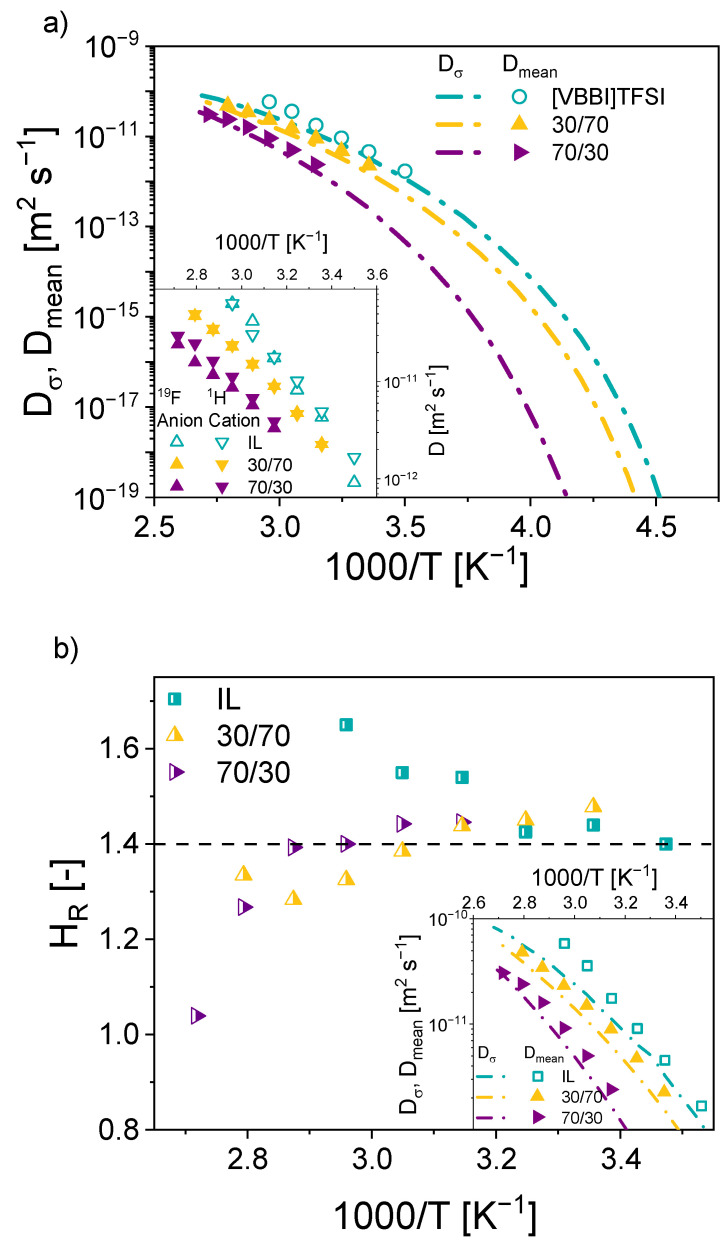
(**a**) Temperature dependence of the mean diffusion coefficients $\left(D_{mean}\right)$ of the IL, 30 wt% polyIL (30/70), and 70 wt% polyIL (70/30) measured by PFG-NMR. The dashed lines represent the diffusion coefficient calculated from BDS $\left(D_{\sigma }\right)$ according to Equation (3). The inset in (**a**) displays the diffusion coefficients of the polycation and anion measured by ^1^H and ^19^F-PFG-NMR, respectively. (**b**) shows the Haven ratio ($H_{R})$ as a function of the temperature for [VBBI]TFSI, 30 wt% polyIL (30/70), and 70 wt% polyIL (70/30). The Haven ratio ($H_{R}$ ) is defined as $D_{mean}$ over $D_{\sigma }$. The inset in (**b**) furthermore depicts the ratio of $D_{mean}$ over $D_{\sigma }$ as quantified by $H_{R}$.

The diffusion coefficients of the cation were slightly higher than the ones of the anion (e.g., 1.8 vs. $2.2\times 10^{-12}$ m^2^ s^−1^ for 70/30 at 313 K) as presented in the inset in Figure 7a. Moreover, the mean diffusion coefficients $\left(D_{mean}=(D_{anion}+D_{cation})/2\right)$ of the samples are displayed and decrease with an increasing amount of polyIL, and therefore decreasing translational mobility and impeding ion transport. To compare $D_{mean}$ with the ionic conductivities observed by BDS, the diffusion coefficient of the conduction process ($D_{\sigma })$ was calculated according to the Nernst-Einstein equation (Equation (3)). The ion diffusion measured by BDS was then correlated with the mean diffusion by PFG-NMR and diffusion coefficients over a wide temperature range of over 100 K were obtained, see Figure 7a.

The temperature dependence of $D_{\sigma }$ follows a VFT form, whereas a clear VFT dependence is not observed for the NMR detected $D_{mean}$ because the temperature range was limited for experimental reasons. At the same temperature $D_{mean}>D_{\sigma }$, which has already been observed for other ILs and their composites [34,36,37]. For ILs, this difference is considered to result from ion pairs and aggregates, which do not contribute to the process of charge transport and can be quantitatively described by the Haven ratio $\left(H_{R}\right)$, see Equation (4). A Haven ration of 1 implies that all ions take part in the charge transport, whereas $H_{R}>1$ indicates the formation of ion pairs or aggregates [34]. In this study, we found $H_{R}$ approx. 1.4 (IL) and 1.45 (blends), indicating that the formation of ion pairs in all three samples is almost temperature independent, see Figure 7b. While $H_{R}$ for IL was previously observed to be in a similar range, the $H_{R}$ of the blends is almost the same, despite the at least four times higher $H_{R}$ of polyILs. The high $H_{R}$ in polyIL is assumed to result from strong ion-ion correlation that hinders ions to take part in the charge transport [35,36]. Thus, our results indicate that the interactions are reduced by the addition of the mobile IL, leading to an enhanced number of ions taking part in the charge transport. This finding might be related to the association of anions to a larger number of polycation chains (e.g., two to four) with the addition of IL resulting in an enhanced number of mobile ions [28]. The deviation at elevated temperatures (around 328 K) is considered to result from charge transport’s domination by thermal energy $\left(k_{B}T\right)$ at higher temperatures, therefore indicating less ion-ion correlation [36]. Moreover, as the $H_{R}$ is found to be similar for both measured blends, we suspect that the ion-ion correlation in the samples is independent of the amount of IL for 30 and 70 wt% polyIL, respectively. This assumption is also corroborated by the plateau of $\epsilon_{s}$ from 70 to 30 wt% polyIL. Additionally, our findings coincide with molecular simulations performed by Mogurampelly et al., who obtained values in the same range and concluded that the dynamical ion correlations of an IL are not influenced by a polyIL [28]. 

Consequently, an increased $\sigma_{0}$ for the blends of polyIL and IL might be caused by the presence of more mobile anions with an increased mobility as a result of decreased ion-ion interactions compared to the pure polyIL. Thus, blending ILs with their corresponding polyIL is a method to obtain homogenous, highly conductive polymeric electrolytes. 

## 4. Conclusions

Herein, we investigated the influence of different blending ratios of poly[VBBI]TFSI as polymerized ionic liquid (polyIL) and the corresponding monomer ionic liquid (IL) [VBBI]TFSI on their ionic conductivity, diffusion, and glass transition temperatures. The IL and polyIL were synthesized and characterized by SEC and NMR. Blends with varying ratios of polyIL to IL were prepared and further investigated via DSC, combined rheo-BDS, and PFG-NMR. The glass transition temperatures $\left(T_{g}\right)$ of the blends were found to obey the Gordon-Taylor equation (Equation (10), k=0.144) being in between the ones of the pure materials. To understand the influence of the increasing amount of IL on the mechanical properties, rheological measurements were performed. The storage modulus $\left(G^{\prime }\right)$ at a certain frequency was found to increase with an increasing amount of polyIL and with a factor $10^{4}$ for the highest (90 wt%) and lowest (10 wt%) amounts of IL at room temperature. Furthermore, BDS measurements revealed that the ionic conductivity $\left(\sigma_{0}\right)$ of the polyIL increased by a factor of $10^{4}$ compared to its monomer at 298 K. Consequently, the ionic conductivities of the blends were also observed to increase, with increasing IL content due to accelerated dynamics and higher content of mobile anions. The temperature dependence of $\sigma_{0}$ followed a Vogel–Fulcher–Tammann (VFT) behavior, which transitions into an Arrhenius behavior for 90 wt% polyIL and the polyIL at the respective $T_{g}$. This decoupling of charge transport from segmental motions is only observed for the blend of 90 wt%, as already 30 wt% of IL led to its suppression. Furthermore, the static dielectric constant $\epsilon_{s}$ was determined to be 10 and 17 for the polyIL and IL, respectively, and the blends showed a plateau at $\epsilon_{s}=12.5$ from 70 to 30 wt% polyIL. The diffusion coefficients of the cation and anion for the IL, 30 wt% polyIL, and 70 wt% polyIL were measured by ^1^H- and ^19^F-PFG NMR, respectively, and a slightly higher diffusion coefficient of cations than of the anions (e.g., 1.8 vs. $2.2\times 10^{-12}$ m^2^ s^−1^ for 70/30 at 313 K) was observed. When the mean coefficients from PFG-NMR were compared to the calculated diffusion coefficients from BDS, the Haven ratio $\left(H_{R},\;\mathrm{see}\;\mathrm{Equation}\;\left(4\right)\right)$ was obtained and quantified. We found $H_{R}$ to be around 1.4 for the IL and 1.45 for 30 wt%, and 70 wt% polyIL at room temperature, and observed a slight decrease for the blends up to 1.25 where the temperature was increased. The value of $H_{R}$ of 1.4 for IL was expected to increase with the addition of polyIL, as $H_{R}>5$ was observed for polyILs by Gainaru et al. [36]. These results show that the blending of the components does not have a strong impact on the charge transport mechanism itself compared to the pure IL. Furthermore, it is highlighted that the increase in the ionic conductivity compared to the pure polyIL might be attributed to the addition of a more mobile phase but also to its ability to reduce the ion-ion correlations in the polyIL. However, the increase in ionic conductivity is achieved to the detriment of the mechanical properties, leading to flowing samples with $G^{\prime \prime }>G^{\prime }$, which are not mechanically strong enough to be applied in actual lithium-ion batteries. Therefore, the next step should cope with the enhancement of the mechanical stability while preserving the high conductivity of the samples with a low wt% of polyIL. 

## Figures and Tables

**Table 1:** Overview of the samples: The names correspond to the mixing ratios of the pure polymerized ionic liquid (polyIL) and ionic liquid (IL) in each weight percent. The glass transition temperature ($T_{g})$ from DSC measurements, the ionic conductivity $\left(\sigma_{0}\right)$ and the storage modulus ($G^{\prime })$ at 298 K and ω=100 rad s^−1^ are displayed.

Sample Name	PolyIL [wt%]	IL [wt%]	$T_{g,DSC}\;[K]{\;}^{1}$	$\sigma_{0,@298K}\;[{S\;\mathrm{cm}}^{-1}]{\;}^{2}$	$G_{@298K}^{\prime }\;{\left[\mathrm{Pa}\right]}^{\;2,3}$
Poly[VBBI]TFSI	100	0	281	$3.4\times 10^{-7}$	$1.4\times 10^{7}$
90/10	90	10	264	$1.3\times 10^{-6}$	$7.2\times 10^{5}$
70/30	70	30	238	$7.7\times 10^{-6}$	$3.3\times 10^{4}$
50/50	50	50	229	$2.9\times 10^{-5}$	$5.8\times 10^{3}$
30/70	30	70	223	$1.1\times 10^{-4}$	$7.5\times 10^{2}$
20/80	20	80	220	$1.4\times 10^{-4}$	$1.8\times 10^{2}$
10/90	10	90	220	$2.2\times 10^{-4}$	$7.5\times 10^{1}$
[VBBI]TFSI	0	100	219	$3.4\times 10^{-4}$	−

^1^ $T_{g}$ was measured by DSC with a heating rate of 10 K min^−1^. ^2^ A graphical summary of $\sigma_{0}$ and $G^{\prime }$ as a function of IL content is displayed in Figure S5. ^3^ The storage modulus was determined at 298 K and ω= 100 rad s^−1^.

**Table 2:** Fit parameters of VFT and Arrhenius fits applied to describe the temperature dependence of the ionic conductivity in Figure 2.

	Vogel–Fulcher–Tammann			Arrhenius	
Sample	$\sigma_{\infty \;}\;[{S\;\mathrm{cm}}^{-1}]$	$B\times 10^{-3}\;\left[K\right]$	$T_{0}\;[K]$	$\sigma_{\infty }^{A}\;[{S\;\mathrm{cm}}^{-1}]$	$E_{a}\;[{\mathrm{kJ}\;\mathrm{mol}}^{-1}]$
Poly[VBBI]TFSI	43.8	22.3	219	$8.1\times 10^{13}$	119
90/10 polyIL/IL	187.8	27.5	208	$3.8\times 10^{19}$	149
70/30 polyIL/IL	239.5	30.3	196	−	−
50/50 polyIL/IL	282.5	31.1	190	−	−
30/70 polyIL/IL	82.6	26.4	189	−	−
20/80 polyIL/IL	97.3	27.3	186	−	−
10/90 polyIL/IL	91.0	26.0	187	−	−
[VBBI]TFSI	70.0	24.6	187	−	−

## Data Availability

Not applicable.

## Supplementary Materials

The following supporting information can be downloaded at: https://www.mdpi.com/article/10.3390/polym14122423/s1, Figure S1. ^1^H-NMR spectrum (400 MHz) of the ionic liquid monomer in deuterated DMSO., Figure S2. ^1^H-NMR spectrum (400 MHz) of the polymerized ionic liquid recorded in deuterated DMSO., Figure S3. Differential scanning calorimetry (DSC) curves of all samples., Figure S4. WAXS data of the neat polyIL, blends, and neat IL. Figure S5. Ionic conductivity $\left(\sigma_{0}\right)$ and storage modulus $\left(G^{\prime }\right)$ as function of ionic liquid content., Figure S6. Determination of the rate of charge transport ($\omega_{c}$) from the real part of the conductivity ($\sigma^{\prime }$) and the imaginary part of the electric modulus (M’’)., Figure S7. Rheological master curves of three different samples with 10 wt% polyIL, 20 wt% polyIL, and 50 wt% polyIL constructed using time-temperature superposition (TTS) at a reference temperature ($T_{ref}$) of 273 K. References [38,46,62] are cited in the supplementary materials.
