# Supplementary material for: Charge Transport and Glassy Dynamics in Blends Based on 1-Butyl-3-vinylbenzylimidazolium Bis(trifluoromethanesulfonyl)imide Ionic Liquid and the Corresponding Polymer
Source: Polymers (Basel). 2022 Jun 15;14(12):2423. doi: 10.3390/polym14122423 (PMC9227190; doi:10.3390/polym14122423)
Supplement: Supplementary file 1 [file polymers-14-02423-s001.zip › polymers-1747663-supplementary.pdf]

Supplementary Information

1. Synthesis

The synthesis of poly[VBBI]TFSI was adapted from Tang et. al [1].

1.1. Synthesis of 1-Butyl-3-vinylbenzylimidazolium chloride ([VBBI]Cl)

13.1 ml 4-vinylbenzylchlorid, 15.4 ml 1-butylimidazole, 30 ml acetonitrile, and 0.1 g 2,6-di-*tert*-butyl-4-methylphenol (BHT) were mixed and stirred under argon at 45 °C for 24 h. The resulting light yellow solution was poured into 400 ml of diethylether and stirred for 30 min. The phases were separated and the viscous phase was washed with diethylether twice. The viscous liquid was dried under reduced pressure and a transparent, very viscous material was obtained. The purity was confirmed by ^{1}H-NMR.

1.2. Synthesis of 1-Butyl-3-vinylbenzylimidazolium bis(trifluoromethanesulfonyl)imide ([VBBI]TFSI)

23.1 g [VBBI]Cl and 0.1 g BHT were dissolved in 50 ml acetone and a solution of 24 g lithium bis(trifluoromethanesulfonyl)imide (LiTFSI) in 50 ml acetone was added slowly. The mixture was stirred at room temperature overnight, filtered and the solvent was removed under reduced pressure (< 50 mbar). The yellow oil was washed with an excess of water, dissolved in acetone, and precipitated in pentane. The phases were separated and the viscous oil was dried under reduced pressure at 50 °C. The purity was confirmed by ^{1}H-NMR and the spectrum is displayed in Figure S1.

1.3. Polymerization of [VBBI]TFSI

2 g [VBBI]TFSI, 2 mg 2,2′-azobis(2-methylpropionitrile) (AIBN), and 2 ml dimethylformamide (DMF) were mixed and stirred at 75 °C for 7 h. The polymer was precipitated in deionized water, dissolved in acetone, and precipitated in deionized water. It was dissolved in methanol and dialyzed against methanol for two days using Spectra/Por® 7 dialysis tubes (Roth, MWCO 1000). The polymer was dried under reduced pressure at 50 °C and the purity was confirmed by ^{1}H-NMR and the spectrum is displayed in Figure S2.

2. Analytical Data

2.1. Nuclear Magnetic Resonance Spectroscopy (NMR)

^{1}H-Nuclear Magnetic Resonance Spectroscopy (NMR) measurements were conducted in deuterated DMSO using a spectrometer at 400 MHz Larmor frequency. The spectra of the ionic liquid monomer and polymerized ionic liquid are displayed and assigned in **Figure S1**.

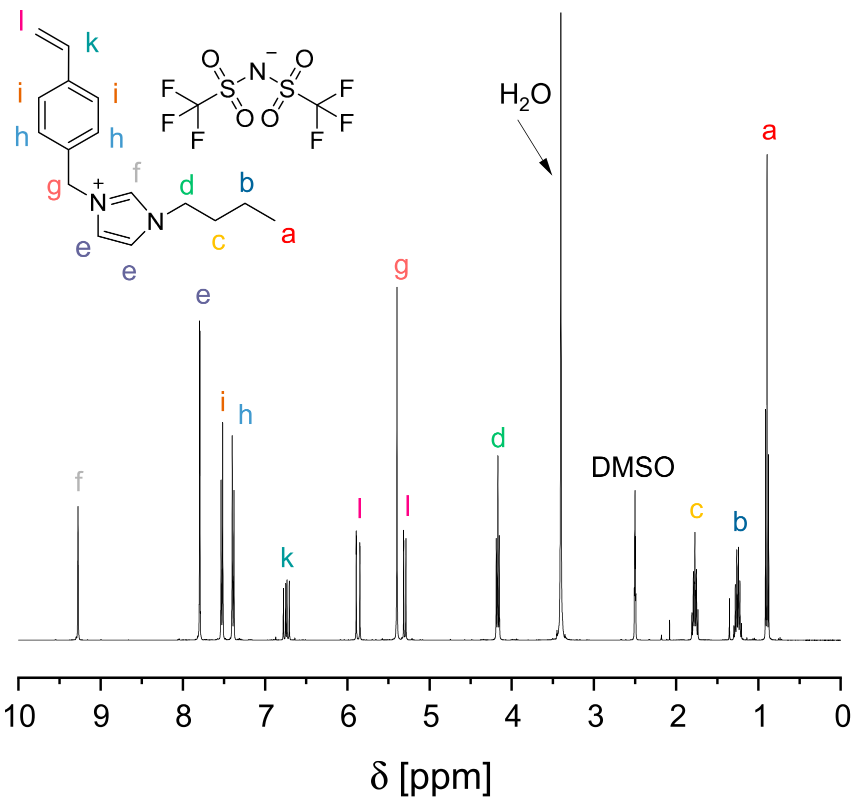

Figure S1. ^{1}H-NMR spectrum (400 MHz) of the ionic liquid monomer in deuterated DMSO.

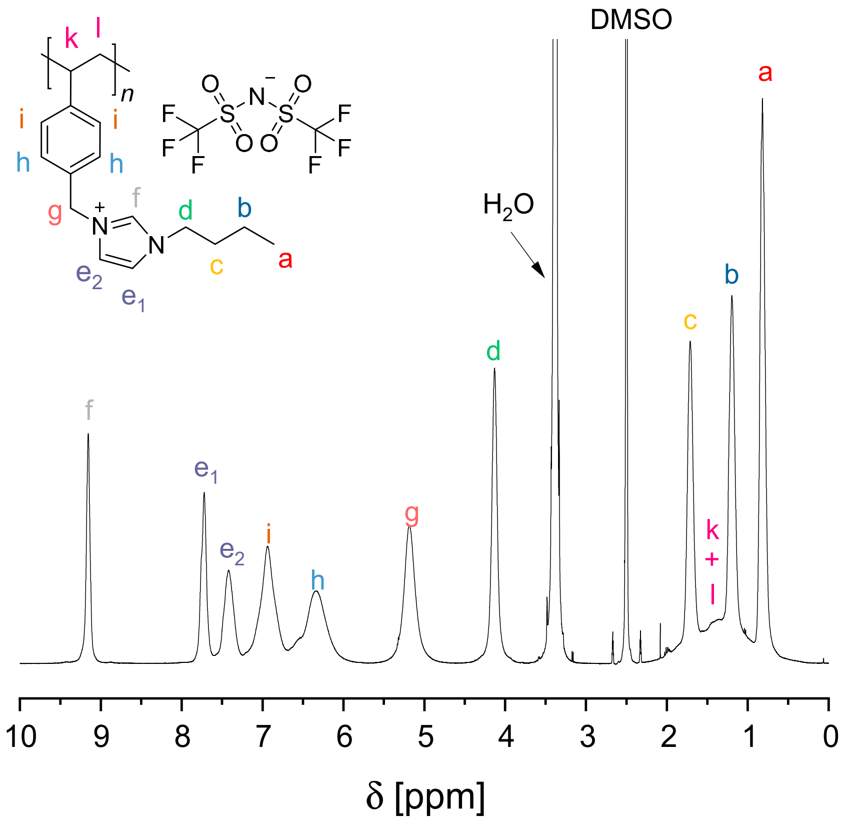

Figure S2. ^{1}H-NMR spectrum (400 MHz) of the polymerized ionic liquid recorded in deuterated DMSO.

2.2. *Differential Scanning Calorimetry (DSC)*

The differential scanning calorimetry (DSC) curves of the second heat cycle with a heating rate of 10 K min^{-1} are displayed in **Figure S3**.

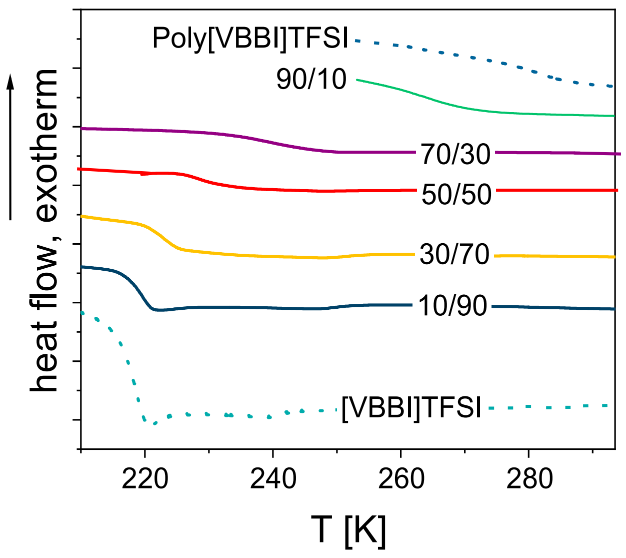

Figure S3. Differential Scanning Calorimetry (DSC) curves of all samples.

2.3. Wide Angle X-Ray Scattering (WAXS)

The WAXS profiles of the polyIL display three unique peaks corresponding to the key correlation length characterizing the morphology of the polyILs. These lengths are related to the scattering wave vector q_x according to Bragg's law ($d_x = 2\pi/q_x$). The samples exhibit scattering peaks at $q_1 = 0.48\ A^{-1}, q_2 = 0.82\ A^{-1}$ *and* $q_3 = 1.33\ A^{-1}$ ($d_1 = 13.09\ A, d_2 = 7.66\ A$ and $d_3 = 4.72\ A$), see **Figure S4**. The higher angle peak q_1 corresponds to the main chain correlation length (backbone-to-backbone), whereas q_2 corresponds to the anion-anion correlations and q_3 to the spatial distance between ionic aggregates [2,3]. The intensity of q_1 and q_2 decreases with the addition of IL proving weaker ionic interactions.

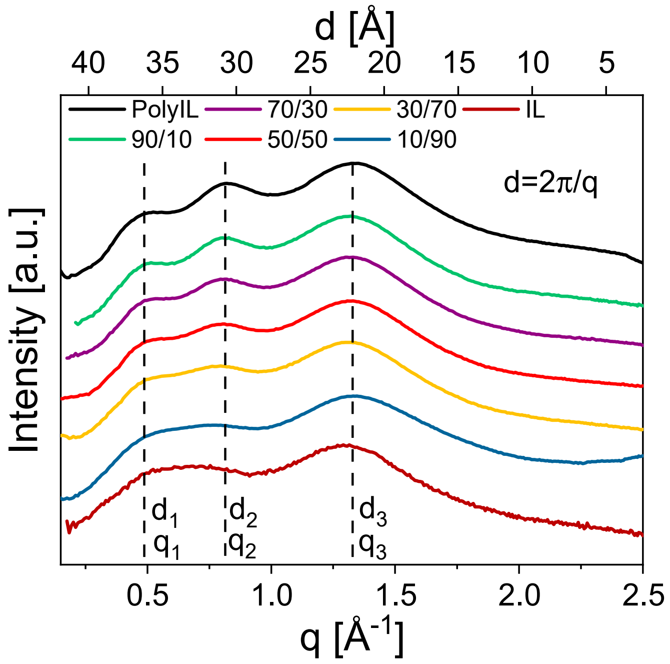

Figure S4. WAXS data of the neat polyIL, blends, and neat IL. The different peaks are indicated by the dotted lines where q_1 corresponds to the chain correlation length (backbone-to-backbone), q_2 to the anion-anion correlations, and q_3 to the spatial distance between ionic aggregates.

2.4. Broadband Dielectric Spectroscopy (BDS) & Rheology

In **Figure S5** the ionic conductivity (σ_0) and the storage modulus (G') at 100 rad s^{-1} as a function of the ionic liquid concentration in the blends are displayed at 298 K. The values behave inversely such as increasing the IL concentration results in increased σ_0 and decreased G'.

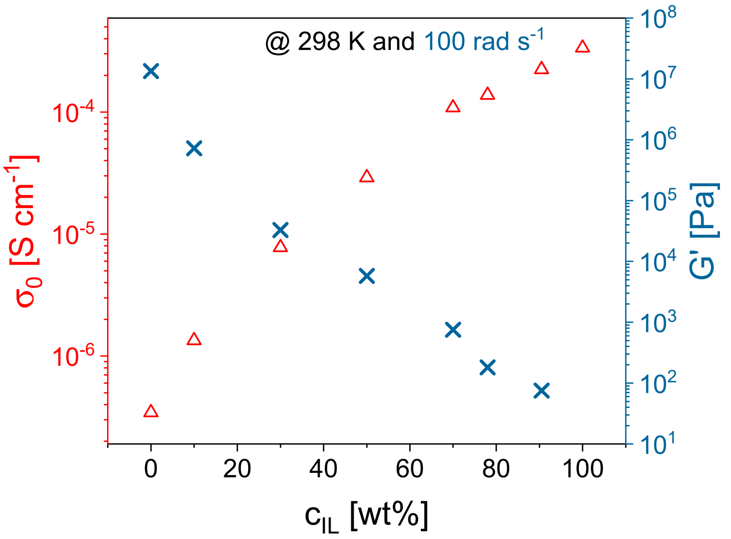

Figure S5. Ionic conductivity (σ_0) and storage modulus (G') as function of ionic liquid content.

Figure S6 displays the close connection of the onset of the ac conductivity in the real part of the conductivity (σ') and the maximum of the imaginary part of the electric modulus (M'').

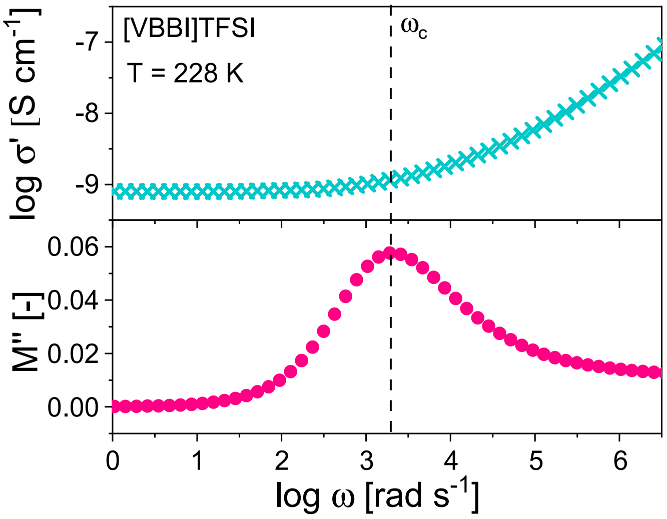

Figure S6. Determination of the rate of charge transport (ω_c) from the real part of the conductivity (σ') and the imaginary part of the electric modulus (M'').

In **Figure S7** the rheological master curves of the remaining samples are shown. The samples with 10 wt% and 20 wt% polyIL show almost the same rheological behavior, whereas for the sample with 50 wt% polyIL an increase in the moduli is observed.

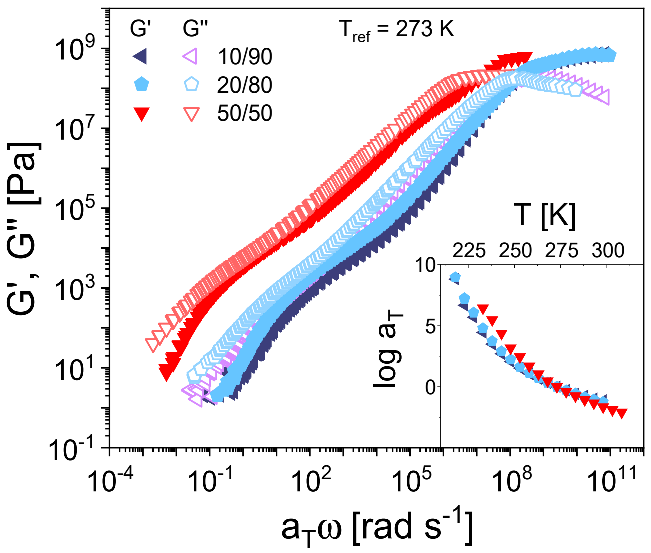

Figure S7. Rheological master curves of three different samples with 10 wt% polyIL, 20 wt% polyIL, and 50 wt% polyIL constructed using time-temperature superposition (TTS) at a reference temperature (T_{ref}) of 273 K.

Publisher's Note: MDPI stays neutral with regard to jurisdictional claims in published maps and institutional affiliations.

References

1. Tang, J.; Tang, H.; Sun, W.; Plancher, H.; Radosz, M.; Shen, Y. Poly(ionic liquid)s: a new material with enhanced and fast CO_2 absorption. *Chem. Commun. (Camb.)* **2005**, 3325–3327, doi:10.1039/b501940k.
2. Choi, U.H.; Ye, Y.; La Salas de Cruz, D.; Liu, W.; Winey, K.I.; Elabd, Y.A.; Runt, J.; Colby, R.H. Dielectric and Viscoelastic Responses of Imidazolium-Based Ionomers with Different Counterions and Side Chain Lengths. *Macromolecules* **2014**, *47*, 777–790, doi:10.1021/ma402263y.
3. Choi, U.H.; Lee, M.; Wang, S.; Liu, W.; Winey, K.I.; Gibson, H.W.; Colby, R.H. Ionic Conduction and Dielectric Response of Poly(imidazolium acrylate) Ionomers. *J. Phys.: Condens. Matter* **2012**, *45*, 3974–3985, doi:10.1021/ma202784e.
